# Structure-guided integrative soft deep clustering analysis of scRNA-seq and scATAC-seq data

**DOI:** 10.3389/fmicb.2025.1678891

**Published:** 2025-09-17

**Authors:** Jiang Xingzuo, Wang Chenyuan, Yao Jiaxi, Wang Chengyuan

**Affiliations:** ^1^Department of Urology, The First Hospital of China Medical University, Shenyang, China; ^2^Department of Epidemiology, School of Public Health, China Medical University, Shenyang, China

**Keywords:** contrastive learning, soft clustering, single-cell clustering, graphlearning, scATAC-seq

## Abstract

**Introduction:**

Current single-cell clustering methods often rely on hard clustering assignments, which fail to capture the dynamic and transitional states of cells during development. This study introduces the Structure-Guided Soft Deep Clustering (sgSDC) framework to address this limitation by integrating multimodal data and enabling probabilistic cluster assignments.

**Methods:**

The sgSDC model combines scRNA-seq and scATAC-seq data using a structure-guided fusion module with global attention. It employs contrastive learning to align modality-specific representations with a consensus representation and introduces a novel soft clustering loss that allows cells to belong to multiple clusters with varying probabilities.

**Results:**

Evaluations on four benchmark datasets demonstrate that sgSDC outperforms eight state-of-the-art methods in Accuracy (ACC), Normalized Mutual Information (NMI), and Adjusted Rand Index (ARI), achieving significant improvements-up to 52.62% in ARI on one dataset.

**Discussion:**

The results validate the effectiveness of structure-guided contrastive learning and soft clustering in capturing cellular heterogeneity. sgSDC provides a robust tool for analyzing complex single-cell data, with potential applications in developmental biology and tumor microenvironment research.

## 1 Introduction

Cells are the fundamental units of life and play pivotal roles in myriad biological functions. With the rapid advancement of single-cell sequencing technologies, data from techniques such as scRNA-seq and scATAC-seq are increasingly accessible (Kashima et al., 2020; Berest and Tangherloni, 2022; Jansen et al., 2019; Yu et al., 2020; Lin et al., 2022), sparking interest among researchers in the differential expression and regulation of characteristics between cells. This interest has now extended to include the joint analysis of both modalities. Multimodal joint analysis not only aids in cell classification and feature identification but also enhances our understanding of cellular developmental processes. Through these cutting-edge techniques, we can explore the intricate networks of cellular functions at the resolution of individual cells, thereby enhancing applications such as genetic diversity analysis and subtyping of cell populations (Yuan et al., 2018; Poulin et al., 2016; Papalexi and Satija, 2018; Zhou et al., 2020; Nguyen et al., 2018). Despite its advantages, single-cell data processing still confronts challenges such as high dimensionality and measurement errors, where the latter can lead to the loss of gene expression information. This loss might be erroneously interpreted as a lack of expression of cellular traits, potentially yielding entirely contrary clinical conclusions in extreme cases.

Advancements in deep learning have ushered in a new paradigm for addressing these challenges, effectively mapping features to low-dimensional spaces and eliminating noise to accurately unveil biological signals. The application of deep learning in computational biology, especially in single-cell data analysis, offers novel perspectives for exploring cellular functions. In the realm of single-cell analysis, deep neural networks, especially autoencoders, have been extensively studied for their capability to extract representations of single-cell data in reduced dimensions (Eraslan et al., 2019; Tran et al., 2021; Yin et al., 2022; Yu et al., 2022). For example, the DCA method (Eraslan et al., 2019) utilizes a negative binomial noise model to improve data quality by considering the count distribution, over-dispersion, and sparsity of data, and demonstrates superiority over existing methods in terms of data recovery and running speed. Additionally, scGMAI (Yu et al., 2021) mitigates information loss by seamlessly integrating data imputation strategies, constructing feature expression matrices crucial for cell-clustering.

After obtaining the feature expression matrices of cells, clustering is considered the most crucial step in the single-cell analysis pipeline, as all subsequent analyses are based on the subgroups defined by clustering. This implies that if the initial cluster categorization is incorrect, subsequent errors will propagate, ultimately rendering the experimental results meaningless. Therefore, the development of accurate and effective clustering algorithms is essential to accurately partition cells based on their feature expression matrices. A significant number of researchers are focused on this area, continuously proposing innovative studies. For instance, techniques such as graph-sc (Ciortan and Defrance, 2022), scASGC (Wang S. et al., 2023), and scGAC (Cheng and Ma, 2022) employ graph autoencoders to transform single-cell data into cell graphs, capturing interactions among cells. Meanwhile, methods such as contrastive-sc (Ciortan and Defrance, 2021), scDCCA (Wang et al., 2023b), and scDECL (Gan et al., 2023) are focused on optimizing autoencoders through contrastive learning, thereby enhancing representation by analyzing similarities and differences between samples. Despite these advances, most existing methods still overlook two critical issues that are essential for effective clustering.

The first issue concerns the integration of information across multiple sequencing results. Most existing algorithms utilize modality-specific encoder networks to learn compressed representations of each type of sequencing result, followed by a rudimentary fusion to achieve what is termed a “consensus representation.” Such brute-force fusion frequently leads to noise and information redundancy, resulting in suboptimal clustering outcomes. To mitigate conflicts between modality-specific private information and shared information, some methods have implemented distinct alignment models. For instance, some researchers have proposed using Kullback-Leibler (KL) divergence to align representation distributions from various sequencing results (Hershey and Olsen, 2007). However, these alignments may not always prove effective, as clusters in scRNA data might correspond with different clusters in scATAC data. Moreover, other researchers have proposed utilizing contrastive learning for data augmentation, yet these methods primarily rely on cell-level samples, treating cell representations of the same cell under different modalities as positive instances and all others as negative. The objective of contrastive learning inherently conflicts with clustering objectives, as such optimization might drive cells away from others within the same cluster. Despite samples within the same cluster are expected to be similar.

**Figure 1:**
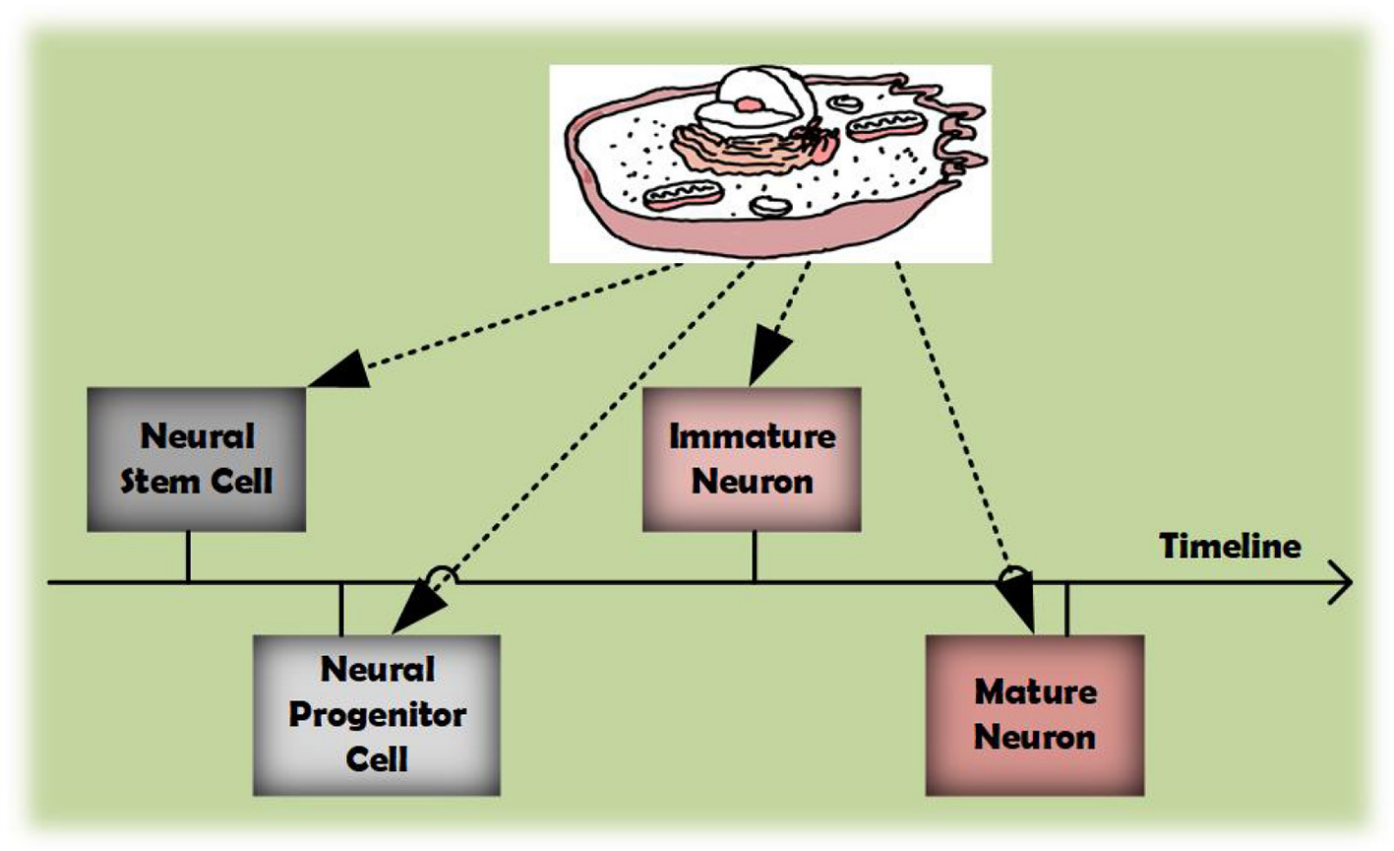
As the timeline progresses, cells can acquire diverse identities during their development.

Another issue pertains to the characteristics of cell data. As demonstrated in Figure 1, as the timeline progresses, the identity of cells can evolve. In clustering tasks, cell identities correspond to cluster labels, indicating that a cell might belong to multiple clusters. Unfortunately, almost all current single-cell clustering algorithms implement hard clustering, where each cell is confined to a single category. For instance, despite scDFC integrating information from multiple dimensions, it restricts a cell to associating with only one cluster. This rigid classification often fails to capture the continuous and transitional states of cellular conditions, leading to suboptimal clustering outcomes. Conversely, soft clustering allows a cell to participate in multiple clusters with varying degrees of membership, thereby offering a more adaptable and accurate classification method. Within the realm of single-cell analysis, soft clustering is often considered a more suitable approach than hard using. Despite this, suitable soft clustering algorithms for multimodal clustering remain largely unexplored.

In response to the previously outlined two issues, we developed the Structure-Guided Soft Deep Clustering (sgSDC) network, a pioneering initiative to apply soft clustering to multimodal single-cell clustering. Specifically, our model is composed of two modules. The first module, the Structure-Guided Information Fusion and Contrastive Learning module, adaptively allocates weights between scRNA and scATAC modalities based on global structural information, and employs contrastive learning to reduce the distance between modality-specific cellular representations and their consensus representation. The second module, the Soft Clustering Optimization module, achieves this by integrating the concept of soft clustering into the traditional KL divergence loss, and develops a novel soft clustering loss function that encourages cells to be assigned to different clusters, thereby optimizing the cellular representations. Empirical evidence confirms the superiority of our proposed algorithm. The core contributions of this work are summarized in three key points:

- We propose the application of soft clustering in the field of single-cell multimodal clustering, achieving high-quality single-cell representations through structure-guided information aggregation and contrastive learning.
- An information fusion method leveraging global structural information has been developed, alongside a contrastive learning approach that aligns modality-specific and consistency representations. Additionally, we have developed a soft clustering loss scheme that allows cells to associate with different clusters with varying probabilities.
- Extensive experiments, encompassing performance comparisons, ablation studies, and parameter sensitivity analyses, have been conducted to confirm the effectiveness of sgSDC against the current state-of-the-art in single-cell multimodal clustering field.

## 2 Materials and methods

The Methods section delineates the sgSDC model in detail, beginning with the Problem Definition to outline the specific mathematical formula in single-cell multimodal clustering. This is followed by Joint Information Aggregation, which explains the effective fusion of information across modalities. Joint Optimization discusses strategies for optimizing the model, and Total Loss Function describes the integration of loss components for enhanced clustering. Model Evaluation presents the three clustering evaluation metrics, and Time Complexity Analysis examines its computational performance, ensuring a holistic understanding of the model's functionality.

### 2.1 Problem definition

**Figure 2:**
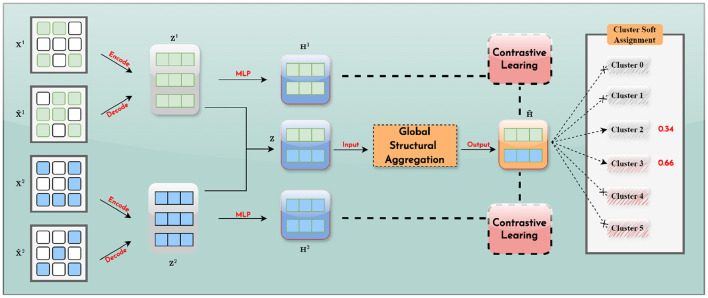
The workflow of sgSDC. It initially integrates information from various modalities, leveraging global structural data to obtain a consensus representation. Subsequently, it utilizes contrastive learning to align each modality-specific representation with the consensus representation. Furthermore, the optimization phase adopts a soft clustering approach, permitting cells to be associated with multiple clusters according to varying probabilities, as illustrated by a 0.34 likelihood of belonging to cluster 2 and a 0.66 probability of association with cluster 3.

The workflow of our proposed sgSDC model is clearly depicted in Figure 2. To ensure clarity for our readers, we first provide mathematical definitions and descriptions of two data types: scRNA and scATAC. Specifically, the data from the scRNA modality is denoted as **X**^1^ and from the scATAC modality as **X**^2^ Algorithm 1. They can be denoted as follows:


(1) $$\begin{array}{l} {\text{X}}^{1}=\left\{{\text{x}}_{1}^{1};\ldots ;{\text{x}}_{n}^{1}\right\}\in {\mathbb{R}}^{n\times d_{1}},\\ {\text{X}}^{2}=\left\{{\text{x}}_{1}^{2};\ldots ;{\text{x}}_{n}^{2}\right\}\in {\mathbb{R}}^{n\times d_{2}}. \end{array}$$

where *d*_1_ represents the feature dimension of the scRNA modality, which indicates the number of features in this modality, while *d*_2_ does the same for the scATAC modality. The single-cell dataset consists of n independent samples, with each containing information from two different modalities: scRNA and scATAC.

**Algorithm 1:** Optimization algorithm of sgSDC.

**Input**: Input data ${\text{X}}^{1}=\left\{{\text{x}}_{1}^{1},\ldots ,{\text{x}}_{n}^{1}\right\}\in {\mathbb{R}}^{n\times d_{1}}$, ${\text{X}}^{2}=\left\{{\text{x}}_{1}^{2},\ldots ,{\text{x}}_{n}^{2}\right\}\in {\mathbb{R}}^{n\times d_{2}}$; The proposed parameters T, α and β; The training iterations I.
1: Preprocess the single-cell data, obtain **X**^1^, and **X**^2^.
2: Aggregate information to build $\hat{H}$ via Equation 6.
3: for *i* = 1 to I **do**
4: Computing the Reconstruction loss using Equation 8;
5: Computing the Contrastive loss using Equation 10;
6: Computing the Soft clustering loss using Equation 12;
7: Optimizing $\hat{H}$ using Equation 14;
8: end **for**
**Output**: Perform *k*-means clustering on $\hat{H}$ to obtain the final results.

Due to the limitations of sequencing data, most publicly available single-cell multimodal datasets currently involve two modalities, and we plan to investigate datasets that encompass more than two modalities in the future. This multimodal data structure enables us to analyze and understand single-cell data from multiple perspectives, thereby potentially increasing the accuracy of clustering analysis through the inclusion of additional information.

### 2.2 Joint information aggregation

Consistent with common practice, we begin our process by compressing features using autoencoders. An autoencoder, a type of unsupervised learning model, compresses features by mapping input data to a lower-dimensional latent space. In our defined biomedical context, we utilize two parallel encoders with respective mapping functions $F_{\theta^{1}}^{1}$ and $F_{\theta^{2}}^{2}$, representing the scRNA and scATAC modalities. Each encoder is configured with its own set of parameters, θ^1^ and θ^2^. The input data **X**^1^ and **X**^2^ are concurrently mapped through these encoders to intermediate representations, as shown below:


(2) $$\begin{array}{l} {\text{Z}}_{i}^{1}=F_{\theta^{1}}^{1}\left({\text{X}}_{i}^{1}\right),\\ {\text{Z}}_{i}^{2}=F_{\theta^{2}}^{2}\left({\text{X}}_{i}^{2}\right). \end{array}$$

where **Z**^1^ and **Z**^2^ respectively denote the cellular representations of scRNA and scATAC. This mapping process facilitates the progressive extraction and compression of critical information within the data, simultaneously eliminating noise and irrelevant details. By preserving essential features and reducing the dimensionality of the data, we significantly enhance the efficiency of subsequent clustering tasks, thereby reducing computational complexity.

Upon completion of feature compression, we concatenate the data from both modalities to form a combined representation. Similarly, we have devised a composite feature transformation matrix **W**_*R*_ to map this combined representation. The mathematical formulation is as follows:


(3) $$\begin{array}{l} \text{Z}=\left[\begin{array}{c} {\text{Z}}^{1}\\ {\text{Z}}^{2} \end{array}\right],\;{\text{W}}_{R}=\left[\begin{array}{c} {\text{W}}_{R1:}\\ {\text{W}}_{R2:} \end{array}\right], \end{array}$$

In the typical feature transformation process, combining **Z** and **W**_*R*_ is usually sufficient. However, this mapping often leads to substantial information redundancy because the elements in **Z** are merely concatenated and not all are considered equally important. Therefore, it is essential to allocate attention according to the global structural information. To initiate this process, we first establish a basic mapping, as outlined below:


(4) $$\begin{array}{l} \left[\begin{array}{c} {\text{R}}_{1:}\\ {\text{R}}_{2:} \end{array}\right]=\left[\begin{array}{cc} {\text{z}}_{1}^{1} & {\text{z}}_{1}^{2}\\ {\text{z}}_{2}^{1} & {\text{z}}_{2}^{2}\\ \vdots & \vdots \\ {\text{z}}_{n}^{1} & {\text{z}}_{n}^{2} \end{array}\right]\left[\begin{array}{c} {\text{W}}_{R1:}\\ {\text{W}}_{R2:} \end{array}\right] \end{array}$$

Next, to allocate attention weights to the representations of various modalities, we must compute a global structural relationship matrix. The dimensions of this matrix **S** correspond to ℝ^*n*×*n*^. The computation process is as follows:


(5) $$\begin{array}{l} \text{S}=\mathrm{softmax}\left(\frac{\text{Z}{\text{W}}_{1}{\left(\text{Z}{\text{W}}_{2}\right)}^{T}}{\sqrt{d}}\right). \end{array}$$

where **W**_1_ and **W**_2_ are trainable matrices specifically designed for additional mappings. *d* represents the unified feature dimension resulting from the transformation. During each information fusion process, the original features are remapped to three distinct spaces. One space is preserved for subsequent use, while the other two are utilized to construct the global structural relationship matrix **S** previously described.

Next, we use **S** ∈ ℝ^*n*×*n*^ to allocate weights to the earlier preserved feature matrix **R**. This process is essentially the product of **S** and **R**. However, if the learned **S** is inaccurate, the performance of the network may significantly deteriorate. To prevent network degradation, we retain the initial features **Z**, and the final form combines **Z** with the product of **S** and **R**, which is then processed through a deep neural network to complete the fusion. The ultimate fused cell representation is denoted as $\hat{H}$ and the computing process is mathematically described as follows:


(6) $$\begin{array}{l} \hat{\text{H}}={\text{W}}_{3}\left(\text{Z}+\overset{\text{n}}{\underset{\text{j}=1}{\sum }}{\text{S}}_{\text{ij}}{\text{R}}_{\text{j}:}\right)+{\text{b}}_{3} \end{array}$$

### 2.3 Joint optimization

After integrating the data representations from scRNA and scATAC sequencing modalities, the resultant consensus representation currently exhibits poor quality and necessitates further optimization. We have meticulously designed three independent optimization loss functions, aiming to significantly enhance the quality of the cell representation through their collaborative effects. These three loss functions are: Reconstruction Loss, Contrastive Loss, and Soft Clustering Loss.

#### 2.3.1 Reconstruction module

Consistent with common practice, the sgSDC network maps the features in the low-dimensional space back to the original feature space. This process ensures that the reconstructed features maintain high consistency with the original features in terms of structure and information. By ensuring the accuracy of the compressed information while eliminating redundancy, the sgSDC network significantly enhances the effectiveness of its compressed features. The mathematical formula to achieve this process is as follows:


(7) $$\begin{array}{l} {\hat{X}}_{i}^{1}=G_{\eta^{1}}^{1}\left(Z_{i}^{1}\right)=G_{\eta^{1}}^{1}\left({ℱ}_{\theta^{1}}^{1}\left(X_{i}^{1}\right)\right),\\ {\hat{X}}_{i}^{2}=G_{\eta^{2}}^{2}\left(Z_{i}^{2}\right)=G_{\eta^{2}}^{2}\left({ℱ}_{\theta^{2}}^{2}\left(X_{i}^{2}\right)\right). \end{array}$$

where $g_{\eta^{1}}^{1}$ and $g_{\eta^{2}}^{2}$ serve as the respective decoders for the scRNA and scATAC modalities. The proposed reconstruction loss is defined below.


(8) $$\begin{array}{l} {ℒ}_{\text{r}}=\sum_{i=1}^{n}{\left\|{\tilde{X}}_{i}^{1}-g_{\eta^{1}}^{1}\left(f_{\theta^{1}}^{1}\left(X_{i}^{1}\right)\right)\right\|}_{2}^{2}\\ \;+\sum_{i=1}^{n}{\left\|{\tilde{X}}_{i}^{2}-g_{\eta^{2}}^{2}\left(f_{\theta^{2}}^{2}\left(X_{i}^{2}\right)\right)\right\|}_{2}^{2}. \end{array}$$

#### 2.3.2 Contrastive module

In single-cell multimodal analysis, the consensus representation $\hat{H}$ must maintain a close alignment with its modality-specific cellular representations **H**^1^ and **H**^2^ within the same cluster. To achieve this, we introduce the powerful approach of contrastive learning. The essence of contrastive learning involves learning the intrinsic structure and feature representations of data by maximizing the similarity between positive sample pairs and minimizing the similarity between negative sample pairs. In our research, we first calculate the similarity between the consensus representation $\hat{H}$ and each modality-specific representation **H**^*m*^. *m* can take two values, either 1 or 2, representing the scRNA and scATAC sequencing modalities associated with **H**^1^ and **H**^2^, respectively. This similarity calculation can be expressed as follows:


(9) $$\begin{array}{l} D\left({\hat{H}}_{i:},H_{i}^{m}\right)=\frac{{\hat{H}}_{i:}^{⊤}H_{i:}^{m}}{\left\|{\hat{H}}_{i:}\right\|\left\|H_{i:}^{m}\right\|},\text{ where }m\in \{1,2\}. \end{array}$$

Building on the similarity outlined above, we further define the structure-guided contrastive loss proposed in this study as follows:


(10) $$\begin{array}{l} {ℒ}_{\text{c}}=-\frac{1}{2n}\sum_{i=1}^{n}\sum_{m=1}^{2}\log\frac{e^{D\left({\hat{H}}_{i:},H_{i:}^{m}\right)/T}}{\sum_{j=1}^{n}e^{(1-S_{ij})D\left({\hat{H}}_{i:},H_{j:}^{m}\right)/T}-e^{1/T}} \end{array}$$

In this formulation, T denotes the temperature hyperparameter as defined in contrastive learning, utilized to control the scale of similarity. **S** represents the global structural relationship matrix. $D\left({\hat{H}}_{i:},{\text{H}}_{i}^{m}\right)$ is the similarity distance as defined previously.

#### 2.3.3 Soft-clustering module

Conventional clustering algorithms require every cell to be classified into a single cluster label, known as hard clustering. In contrast, soft clustering permits a data point to belong to multiple coarse labels simultaneously. Before proceeding, we introduce the most common clustering loss function, as follows:


(11) $$\begin{array}{l} L_{\text{Kullback}-\text{Leibler}}=\underset{i}{\sum }\underset{j}{\sum }p_{ij}\log\frac{p_{ij}}{q_{ij}}, \end{array}$$

The KL divergence loss, as mentioned above, is widely used in various deep single-cell clustering studies. Its underlying principle involves calculating *q*_*ij*_ using the Student's t-distribution. Subsequently, the target distribution *p*_*ij*_ is derived from *q*_*ij*_, and the KL divergence loss is applied to minimize the distance between *q*_*ij*_ and *p*_*ij*_. This approach enhances the quality of the representation.

In our design, to align with the soft clustering characteristics exhibited during cellular development, we innovatively replace the conventional *p*_*ij*_ with γ_*ij*_ to construct the pillar of the scientific debate and the protocol framework as defined in our study, as follows:


(12) $$\begin{array}{l} L_{\text{s}}=\underset{i}{\sum }\underset{j}{\sum }\gamma_{ij}\log\frac{\gamma_{ij}}{q_{ij}}. \end{array}$$

Where γ_*ij*_ represents the probability distribution for soft clustering, calculated by optimizing the following soft clustering objective, expressed as follows:


(13) $$\begin{array}{c} \underset{\gamma_{ij}}{\min}\sum_{j=1}^{k}\gamma_{ij}^{m}\|{\tilde{z}}_{i}-\mu_{j}{\|}^{2},\\ s.t.\;\sum_{j=1}^{k}\gamma_{ij}=1, \end{array}$$

This objective entails the minimization of the weighted distance, where the weighting factor γ_*ij*_ accounts for the degree of membership of each data point to the cluster centers. The exponent *m* amplifies the penalty for clusters with lower degrees of membership, thereby enhancing the robustness of the algorithm. It is a real number greater than 1 known as the controlling index, modulates the degree of soft assignment in the clustering. *k* denotes the total number of clusters, ${\tilde{z}}_{i}$ represents the *i*-th data point, μ_*j*_ the center of the *j*-th cluster.

### 2.4 Total loss function

Given the proposed sgSDC model, which incorporates three parallel loss functions: reconstruction loss, contrastive loss, and soft clustering loss, we have introduced two additional hyperparameters, α and β, into the overall loss function to control the weight of each loss component. This facilitates optimal tuning of the model's performance. Consequently, the total loss function can be expressed as follows:


(14) $$\begin{array}{l} ℒ={ℒ}_{r}+\alpha {ℒ}_{c}+\beta {ℒ}_{s} \end{array}$$

### 2.5 Model evaluation

Three widely utilized clustering evaluation metrics are used to assess the model, specifically: Accuracy (ACC), Normalized Mutual Information (NMI), and Adjusted Rand Index (ARI), their definitions are provided below. ACC is constructed to measure the correctness of classification. It is defined as follows:


(15) $$\begin{array}{l} \text{ACC}=\frac{\sum_{i=1}^{n}I\left(y_{i}={ŷ}_{i}\right)}{n}. \end{array}$$

NMI is built on the degree of information shared between the clusters and the true classifications. It is defined as follows:


(16) $$\begin{array}{l} \text{NMI}=\frac{2MI(U,V)}{H(U)+H(V)}, \end{array}$$

ARI is constructed based on the similarity between the clustering result and the ground truth. It is defined as follows:


(17) $$\begin{array}{l} \text{ARI}=\frac{\sum_{ij}\left(\begin{array}{c} n_{ij}\\ 2 \end{array}\right)-[\sum_{i}\left(\begin{array}{c} a_{i}\\ 2 \end{array}\right)\sum_{j}\left(\begin{array}{c} b_{j}\\ 2 \end{array}\right)]/\left(\begin{array}{c} n\\ 2 \end{array}\right)}{\frac{1}{2}[\sum_{i}\left(\begin{array}{c} a_{i}\\ 2 \end{array}\right)+\sum_{j}\left(\begin{array}{c} b_{j}\\ 2 \end{array}\right)]-[\sum_{i}\left(\begin{array}{c} a_{i}\\ 2 \end{array}\right)\sum_{j}\left(\begin{array}{c} b_{j}\\ 2 \end{array}\right)]/\left(\begin{array}{c} n\\ 2 \end{array}\right)}, \end{array}$$

### 2.6 Time complex analysis

The time complexity of the sgSDC model is given by $O\left(\overset{2}{\underset{m=1}{\sum }}n^{2}d_{m}I+\overset{2}{\underset{m=1}{\sum }}nd_{m}^{2}I+\overset{2}{\underset{m=1}{\sum }}nd_{m}I\right)$, where I represents the iterations of the training process. Specifically, the computational cost associated with dimension reduction during training is $O\left(\overset{2}{\underset{m=1}{\sum }}nd_{m}I\right)$, and for the information fusion module, it is $O\left(\overset{2}{\underset{m=1}{\sum }}n^{2}d_{m}I+\overset{2}{\underset{m=1}{\sum }}nd_{m}^{2}I\right)$. The contrastive learning module incurs a cost of $O\left(\overset{2}{\underset{m=1}{\sum }}n^{2}d_{m}I\right)$. From the perspective of time complexity, the algorithm is closely associated with the quadratic term of *n*, which implies that the time complexity will increase quadratically as *n* increases.

## 3 Experiments

We have meticulously designed a suite of comprehensive experiments aimed at thoroughly assessing the performance of our model. To ensure the logical progression of our research, our experiments are organized to address the following four key research questions (RQ): (1) Does sgSDC outperform other state-of-the-art methods in the context of single-cell deep clustering? (2) Is the contrastive learning strategy proposed by sgSDC effective? (3) Is the soft clustering strategy proposed by sgSDC effective? (4) Does the performance of sgSDC vary significantly with different hyperparameters?

### 3.1 Experimental settings

#### 3.1.1 Resources for benchmark datasets and preprocessing

As shown in Table 1, four publicly available single-cell benchmark datasets were used to evaluate the proposed software in our study. Some datasets were already processed; thus, no further processing was performed. For those without prior quality control, we selected the top 2,000 features using the Scanpy package. Additionally, the links to the data resources are listed below:

- **D1:** https://www.ncbi.nlm.nih.gov/geo/query/acc.cgi?accGSE128639
- **D2:** https://www.10xgenomics.com/resources/datasets
- **D1:** https://github.com/YosefLab/totalVI_reproducibility
- **D4:** https://www.10xgenomics.com/resources/datasets

**Table 1:** Benchmark multi-modal datasets include scRNA-seq and scATAC-seq data.

**Dataset**	**Cell**	**Sequencing**	**N_Clusters**	**Dimension**
D1	1,728	2	5	[1,000, 25]
D2	3,762	2	16	[1,000, 49]
D3	6,018	2	10	[1,000, 112]
D4	2,585	2	14	[2,000, 2,000]

#### 3.1.2 Baseline methods

We compared sgSDC with eight competitive methods, chosen for their foundational significance, recent contributions, or extensive citation metrics, as representative approaches in the field. The specific details are outlined below:

- **k-means:** “Some Methods for Classification and Analysis of Multivariate Observations” (MacQueen et al., 1967)
- **Spectral Clustering:** “A Tutorial on Spectral Clustering” (Von Luxburg, 2007)
- **FastMICE:** “Fast Multi-View Clustering Via Ensembles: Toward Scalability, Superiority, and Simplicity” (Huang et al., 2023)
- **EEOMVC:** “Structured Graph Learning for Scalable Subspace Clustering: From Single View to Multiview” (Wang et al., 2023a)
- **AMGL:** “Parameter-Free Auto-Weighted Multiple Graph Learning: A Framework for Multiview Clustering and Semi-Supervised Classification” (Nie et al., 2016)
- **OMVFC:** “Latent information-guided one-step multi-view fuzzy clustering based on cross-view anchor graph” (Zhang et al., 2024)
- **scEMC:** “Effective multi-modal clustering method via skip aggregation network for parallel scRNA-seq and scATAC-seq data” (Hu et al., 2024)
- **scMVAE:** “Deep-joint-learning analysis model of single cell transcriptome and open chromatin accessibility data” (Zuo and Chen, 2021)

#### 3.1.3 Training details

The experimental environment was established on a server running Ubuntu 22.04 LTS, capable of optimally utilizing the machine's performance. The hardware specifications include a CPU: Intel Core i7-6800K, 64GB of DDR4 memory, and a NVIDIA TITAN Xp graphics critical. Regarding network parameters, the bottleneck layer was set to 64, and the dimension resulting from the fusion of two modalities was established at 128. The soft clustering control coefficient m was set at 1.5. The network underwent 200 rounds of pre-training followed by 50 rounds of training. An early stopping mechanism was implemented, halting the training if there was no improvement over 20 epochs. The learning rate was set at 0.0005. The Python version employed was 3.7, and the Pytorch version was 1.13.1.

### 3.2 Comparison results cross four benchmark datasets (RQ1)

**Figure 3:**
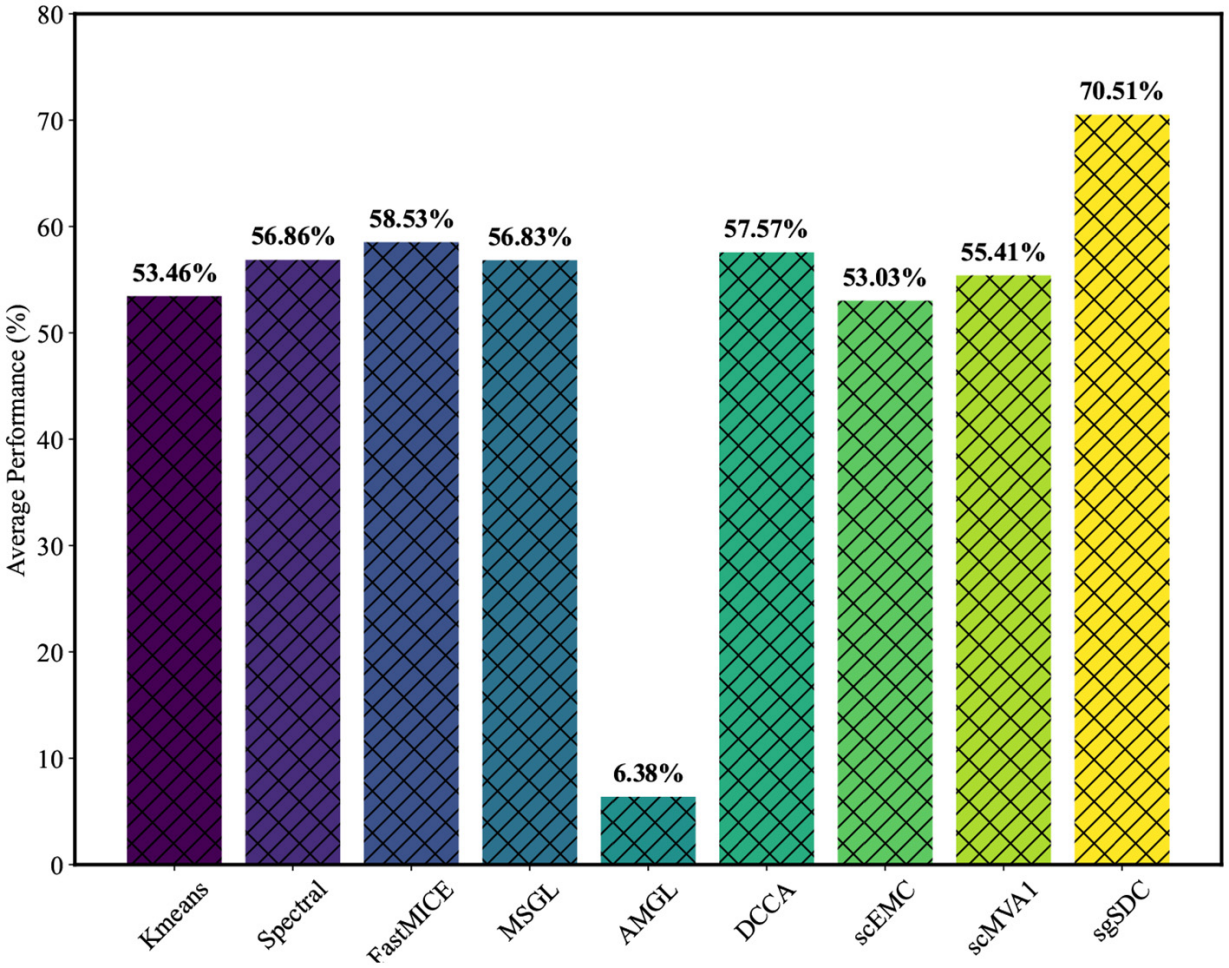
The average performance of various baseline methods on the datasets D1, D2, D3, and D4, measured on three metrics, clearly demonstrates which method is superior.

sgSDC is a soft clustering, multimodal algorithm designed specifically for the characteristics of single-cell data. In this section, we systematically evaluate its performance in clustering tasks. Specifically, we compare sgSDC with the eight baseline methods introduced earlier, and Table 2 presents the results on four real scRNA-seq and scATAC multimodal datasets. These results are recorded under optimal parameter settings. The conclusions of the study are clear: sgSDC consistently achieves competitive ACC, NMI, and ARI scores compared to the baseline methods. To illustrate this more intuitively, we highlight the best results in blue and underline the second-best results. Notably, sgSDC achieved ten first-place finishes across three metrics on the four datasets, demonstrating its stable and superior clustering performance in various scenarios. Compared to the next best results, the improvements in clustering performance are significant, with increases of 20.44%, 13.87%, and 52.62% on D1; 2.82% and 3.38% on D2; and 7.95%, 11.47%, 10.60%, 0.73%, and 36.29% on D3 and D4. To more vividly illustrate the comparative nature of the experimental outcomes, the average values of the results in the table were computed, and the visualized outcomes are displayed in Figure 3.

**Table 2:** The comparison results among sgSDC and eight baseline methods are presented.

**Dataset**	**Metric**	**Kmeans**	**Spectual**	**FastMICE**	**EEOMVC**	**AMGL**	**OMVFC**	**scEMC**	**scMVAE**	**sgSDC**
D1	ACC	0.6389	0.7240	0.7862	0.8096	0.2168	0.7674	0.6458	0.6542	0.9468
	NMI	0.6726	0.6478	0.7560	0.7958	0.3200	0.6955	0.6645	0.6819	0.8609
	ARI	0.4477	0.4450	0.5877	0.6395	0.0004	0.5432	0.4354	0.4562	0.8968
D2	ACC	0.5811	0.6146	0.6623	0.6768	0.0850	0.6223	0.5534	0.6135	0.6810
	NMI	0.6095	0.6255	0.7019	0.7006	0.0130	0.6760	0.6718	0.6850	0.6959
	ARI	0.4349	0.4301	0.5613	0.5243	0.0001	0.5062	0.4159	0.5195	0.5803
D3	ACC	0.5150	0.6143	0.4923	0.6407	0.1165	0.6073	0.5452	0.5288	0.6632
	NMI	0.6622	0.6886	0.6525	0.6605	0.0066	0.6881	0.5295	0.6678	0.6141
	ARI	0.4338	0.5387	0.4150	0.5413	0.0005	0.5658	0.2743	0.4561	0.6005
D4	ACC	0.4418	0.5176	0.4793	0.5691	0.0932	0.6588	0.6050	0.4569	0.6692
	NMI	0.5498	0.5508	0.5601	0.5378	0.0183	0.6025	0.6122	0.5394	0.6167
	ARI	0.3434	0.3589	0.3244	0.4053	0.0002	0.5153	0.4352	0.3008	0.5932

The best results are highlighted in red, while the runner-up results are underlined.

On the other hand, the experimental results indicate that the EEOMVC method, which employs a unified one-step strategy, performed well and is particularly suited to single-cell scenarios, warranting further exploration. Although most algorithms achieved decent performance, the AMGL algorithm exhibited extremely poor clustering performance. This graph-based model struggles with the complexity of biological environment signals, making it nearly impossible to construct an accurate cell-to-cell graph. Therefore, AMGL's poor clustering performance may result from incorrect cell graphs. In summary, although no universal clustering algorithm exists, sgSDC has demonstrated significant improvements in all aspects compared to existing algorithms.

### 3.3 Ablation study of the contrastive learning module (RQ2)

The SGSDC model features an innovative structure-guided contrastive learning module, meticulously designed to narrow the discrepancies between modality-specific representations and a unified consensus representation. To ascertain the validity of this innovative module, we embarked on comprehensive ablation experiments focusing on the contrastive learning component. Specifically, we strategically eliminated the custom-designed contrastive loss to gauge its impact on the model's overall performance.

**Figure 4:**
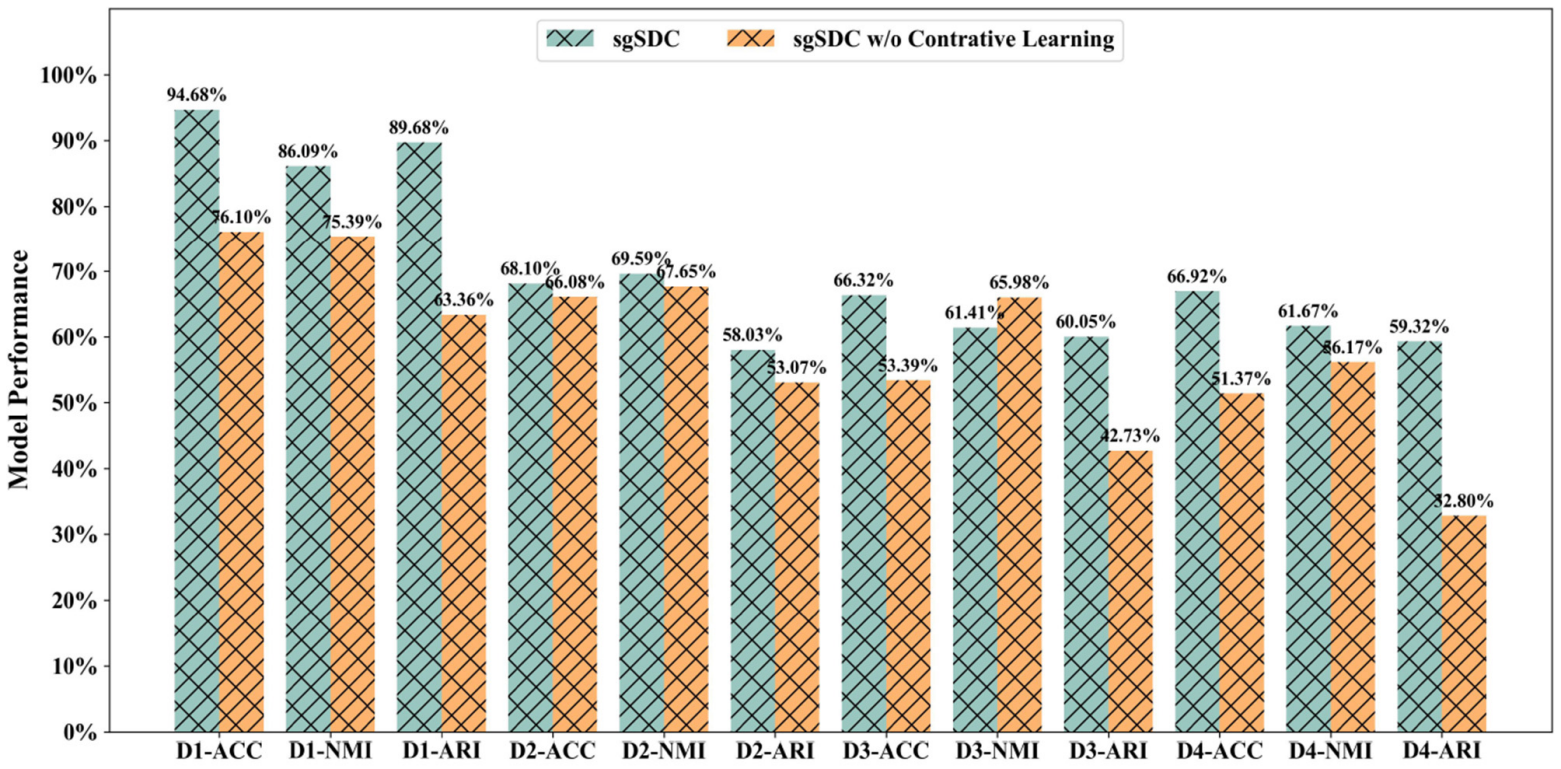
The designed ablation study for the contrastive learning module to validate it effectiveness, the abscissa displays the datasets and metrics, while the ordinate lists the corresponding values of the metrics.

The outcomes, graphically represented in Figure 4, clearly illustrate a marked decline in performance following the omission of the contrastive learning module. This substantiates the pivotal role of our contrastive learning component in effectively bridging the disparities between modality-specific representations and the consensus representation, thereby mitigating the adverse effects of information redundancy and conflicting data on the clustering performance. In conclusion, the ablation studies outlined in this section robustly reinforce the efficacy and critical importance of the proposed contrastive learning strategy. Although the method employed for selecting positive and negative samples in this investigation remains relatively rudimentary, future endeavors could focus on devising more advanced selection algorithms to further enhance the outcomes.

### 3.4 Ablation study of the soft clustering module (RQ3)

As previously postulated, soft clustering may indeed prove to be a more appropriate strategy for single-cell clustering applications. Nevertheless, these propositions remain speculative; therefore, in this section, we aim to rigorously assess the efficacy of soft clustering algorithms through structured empirical testing. We conducted ablation experiments specifically targeting the soft clustering component, developing an alternative version of the sgSDC model that omits the soft clustering methodology. By comparing the clustering performance of this modified variant with the complete sgSDC model, we have collected valuable insights concerning the relevance and potential benefits of soft clustering in biomedical settings.

**Figure 5:**
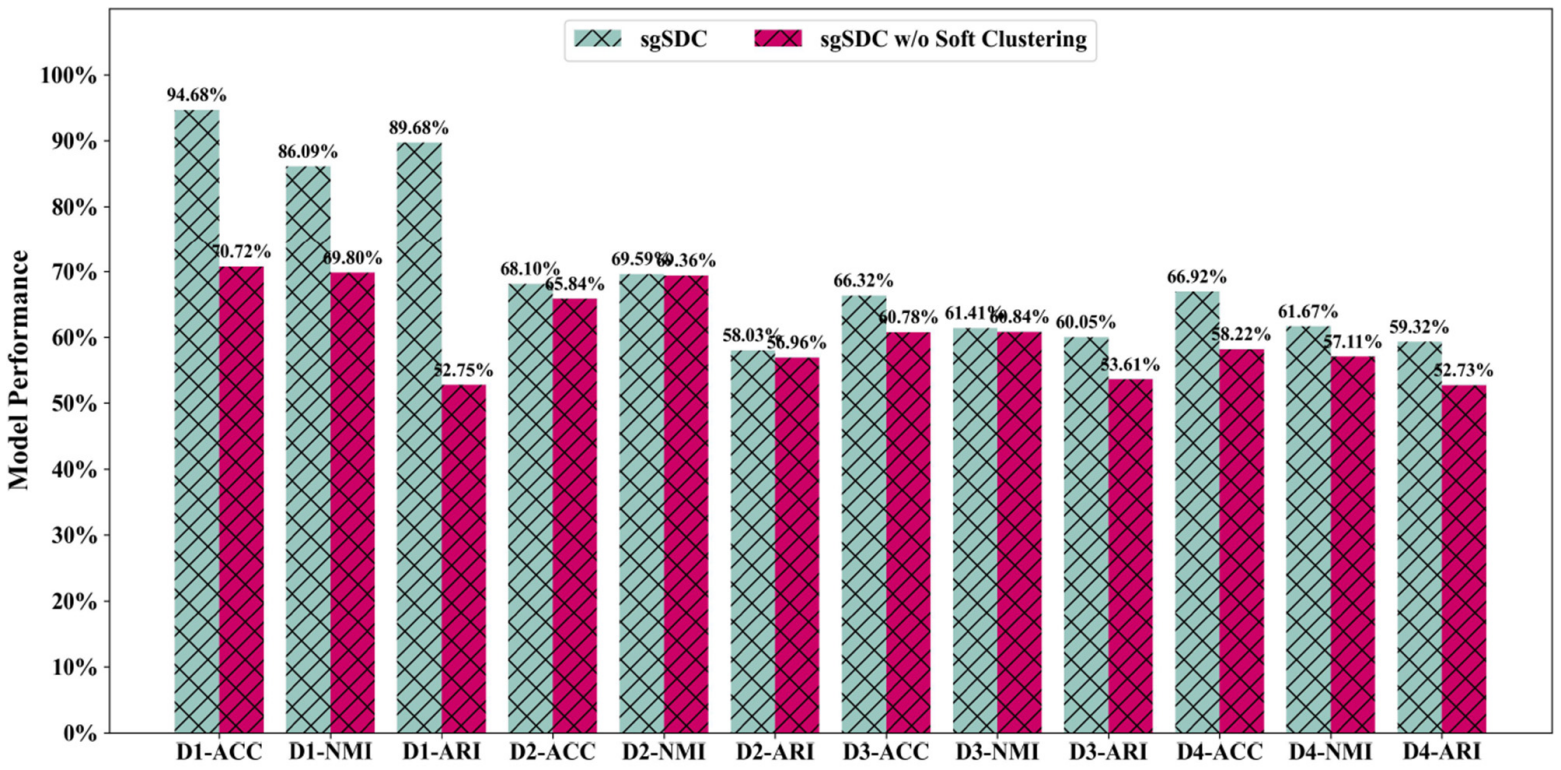
The designed ablation study for the soft clustering module to validate it effectiveness, the abscissa displays the datasets and metrics, while the ordinate lists the corresponding values of the metrics.

The empirical outcomes, as illustrated in Figure 5, clearly reveal a marked deterioration in the clustering capabilities of the sgSDC variant devoid of the soft clustering approach (sgSDC w/o soft). The omission of this strategy not only diminishes the model's performance but also markedly impacts its operational efficacy. Consequently, the data from these experiments substantiate the effectiveness of the soft clustering approach, unequivocally affirming its superiority over traditional hard clustering techniques in the specialized realm of single-cell analysis.

### 3.5 Parameter analysis (RQ4)

During the training phase of the sgSDC model, we defined two sets of hyperparameters. Experiments will be conducted to observe the sensitivity of these parameters under various combinations.

#### 3.5.1 Investigation of trade-off parameter α and β

**Figure 6:**
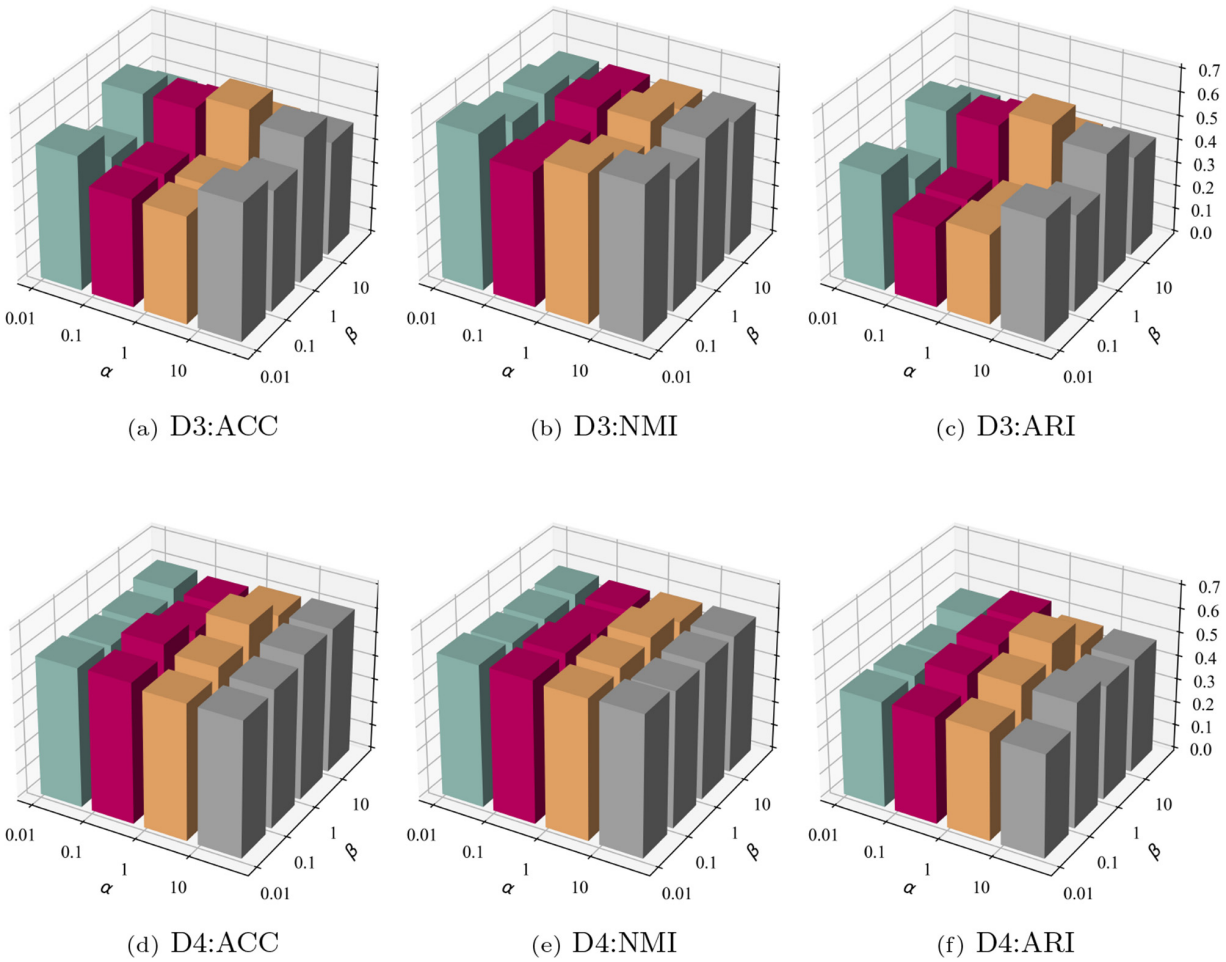
Parameter sensitivity analysis on datasets D3 and D4. The top row **(a–c)** shows clustering performance (ACC, NMI, ARI) on D3, while the bottom row **(d–f)** depicts the same metrics for D4. Each plot illustrates the impact of varying hyperparameters α and β on model performance.

In the introduction of the optimization module for sgSDC, three loss functions were proposed to jointly optimize cell representations. Determining the trade-off among these three loss functions is challenging. Consequently, the impact of the trade-off parameters α and β on the model's performance was investigated. Specifically, an exploration space of {0.01, 0.1, 1, 10} was defined for both parameters, yielding 16 sets of outcomes. To enhance the presentation of these findings, the results were visualized in a three-dimensional graph as illustrated in Figure 6. From the experimental results, the following conclusions can be inferred: (1) The sensitivity of parameters α and β varies across datasets; for instance, they exhibit sensitivity on dataset D3 but not on D4. (2) In the majority of cases, the model demonstrates leading performance when β is set to 1. (3) The sensitivity of β is greater than that of α, suggesting that the proposed soft clustering loss significantly impacts the model, leading to noticeable fluctuations as its coefficient varies.

#### 3.5.2 Investigation of temperature parameter T

In the contrastive learning module, a temperature parameter was introduced to control the scaling. The parameter space {0.1, 0.3, 0.5, 0.7, 0.9} was traversed to investigate the impact of this hyperparameter on the model's performance, with all other parameters held constant. According to Table 3, setting the temperature parameter to 0.5 results in optimal performance for the model in most scenarios. Consequently, it is recommended to set the temperature parameter T at 0.5 due to its sensitivity.

**Table 3:** Investigation of the temperature parameter T on model's clustering performance.

**Datasets**	** T **	**ACC**	**NMI**	**ARI**
D1	0.1	0.7350	0.6496	0.5274
	0.3	0.7245	0.6480	0.5080
	0.5	0.9468	0.8609	0.8968
	0.7	0.7303	0.7083	0.5657
	0.9	0.7355	0.7108	0.5742
D2	0.1	0.6411	0.6625	0.5566
	0.3	0.6353	0.6745	0.5334
	0.5	0.6810	0.6959	0.5803
	0.7	0.6337	0.6815	0.5625
	0.9	0.5691	0.5990	0.4449
D3	0.1	0.4787	0.6134	0.3639
	0.3	0.4924	0.5859	0.4087
	0.5	0.6632	0.6141	0.6005
	0.7	0.4718	0.6170	0.3880
	0.9	0.4809	0.6170	0.3896
D4	0.1	0.5919	0.5769	0.5413
	0.3	0.5938	0.5819	0.4935
	0.5	0.6692	0.6167	0.5932
	0.7	0.6039	0.5776	0.5334
	0.9	0.5667	0.5710	0.4268

The runner-up results are underlined.

## 4 Conclusion

In conclusion, we introduce a novel structure-guided single-cell multimodal soft clustering algorithm, sgSDC, that achieves more accurate cellular cluster delineation. This model effectively integrates cross-modal information through the synergistic operation of its components, eliminating redundancy across modalities and facilitating soft assignments of cellular clusters. Specifically, we assign different weights to each modality during the aggregation process based on a global attention mechanism, then use contrastive learning to align modality-specific representations with a consistent representation, ultimately obtaining a clustering-friendly cellular representation. Additionally, we employ an innovative soft clustering strategy to model the single-cell scenario, which aligns closely with the real-world characteristics of single-cell data. Comprehensive experimental validation confirms sgSDC's superiority, and ablation studies underscore the effectiveness of each module.

### 4.1 Limitations of the study

This work also faces limitations due to sequencing technology constraints, as datasets larger than two modalities are still scarce; thus, our experiments were limited to bimodal datasets. The current limitation of sgSDC to bimodal datasets may restrict its generalizability to emerging multimodal technologies that simultaneously capture transcriptomics, proteomics, and spatial data. This could hinder applications in complex biological systems where three or more modalities are needed to fully resolve cellular states—for instance, in tumor microenvironments requiring joint analysis of gene expression, surface proteins, and chromatin accessibility. To address this, future work will expand sgSDC's architecture to n-modality integration by: developing a hierarchical attention mechanism to dynamically weight additional modalities, and incorporating modality-specific batch correction layers to handle technical variability across platforms. Furthermore, the optimization function for soft clustering can be further refined for improved performance. In the future, we plan to expand the contrastive learning module and refine the strategy for selecting positive and negative samples to better accommodate complex biological data distributions.

## Data Availability

The raw data supporting the conclusions of this article will be made available by the authors, without undue reservation.
